# Mediation analysis for logistic regression with interactions: Application of a surrogate marker in ophthalmology

**DOI:** 10.1371/journal.pone.0192857

**Published:** 2018-02-12

**Authors:** Signe M. Jensen, Hanne Hauger, Christian Ritz

**Affiliations:** 1 Department of Plant and Environmental Sciences, University of Copenhagen, Højbakkegård Allé 13, DK-2630 Taastrup, Denmark; 2 Department of Nutrition, Exercise and Sports, University of Copenhagen, Rolighedsvej 26, DK-1958 Frederiksberg C, Denmark; University of Texas School of Public Health, UNITED STATES

## Abstract

Mediation analysis is often based on fitting two models, one including and another excluding a potential mediator, and subsequently quantify the mediated effects by combining parameter estimates from these two models. Standard errors of such derived parameters may be approximated using the delta method. For a study evaluating a treatment effect on visual acuity, a binary outcome, we demonstrate how mediation analysis may conveniently be carried out by means of marginally fitted logistic regression models in combination with the delta method. Several metrics of mediation are estimated and results are compared to findings using existing methods.

## Introduction

In a randomized clinical trial in ophthalmology the effect of an experimental treatment, interferon-*α*, on loss of vision in patients with age-related macular degeneration was compared to a placebo treatment [1, 2]. Age-related macular degeneration is a medical condition of irreversable vision loss in the center of the visual field and only a small minority of patients are amendable for laser treatment. Loss of vision was assessed as loss of the ability of reading lines of letters from standardized vision charts. The main outcome considered was whether or not the patient had lost at least three lines of vision one year after baseline assessment. A secondary outcome that may be thought of as a mediator was whether or not at least two lines of vision were lost 6 months after baseline.

Mediation analysis is a widely used methodology in behavioural and social sciences as well as psychology. One common paradigm, referred to as the single-mediator approach, is to fit two models to the same outcome, both including the effect of interest (e.g., a treatment contrast) but either including or excluding a potential mediator and subsequently quantify the mediated effect by combining parameter estimates from the two marginal model fits [3]. Specifically, the difference or the proportion of the effect of interest, which is mediated, is then estimated as a derived parameter using parameter estimates from both model fits. For linear models the approximate standard error of the mediated effect has been derived explicitly using the delta method [4, 5]. For more complex marginal model fits both the normality-based or simulation-based delta method [6, 7] is still feasible if the correlations between parameter estimates in different models can be provided. Alternatively, bootstrap techniques may be used [8]. However, for more complex models such as generalized linear models and linear mixed models, these approaches become much more computationally intensive. Yet another possibility would be to formulate a joint model [9], but such joint models may also be challenging and to our knowledge have not been proposed in the context of mediation analysis.

The aim of the present paper is to demonstrate how to use marginal logistic regression model fits and sandwich variance estimators, as originally proposed by White (1982) [10], in combination with the delta method for carrying out mediation analysis in ophthalmology. Specifically, the interest was in how much of the treatment effect on visual acuity at one year was mediated through visual aquity at six months [1, 11]. In this case, where logistic regression has to be used, the proportion mediated may be defined in several ways but it is always found by combining parameters from two or three regression models.

## Methods

First, we briefly define the statistical model and the key concepts of mediation analysis before returning to the application to ophthalmology.

Let (*y*_1_, …, *y*_*N*_) be a random vector of mutually independent binary observations. We will assume that the expectation of *y*_*i*_ may be described by:

$$\begin{array}{c} E\left(y_{i}\right)=g\left(x_{i},\beta \right) \end{array}$$

for some known linear or nonlinear function $g:\;R^{p}\;\times \;R^{q_{1}}\;\mapsto \;R$ that maps the *p*-dimensional covariate space (with elements *x*_*i*_) and the *q*_1_-dimensional parameter space (with elements ***β***) into a subset of the real axis. The variance of *y*_*i*_ may (or may not) be a function of covariates and/or the expectation, depending on the distribution assumed for the data:

$$\begin{array}{c} \text{var}\left(y_{i}\right)=h\left(x_{i},\beta ,\sigma \right) \end{array}$$

where ***σ*** denotes a *q*_2_-dimensional vector of variance parameters. Let ***θ*** = (***β***, ***σ***) be the *q*_1_ + *q*_2_-dimensional vector of all parameters in the model.

Parameter estimates are readily obtained using maximum likelihood estimation. If the assumed model is misspecified, in the sense that the data are generated from a distribution that is not among the distributions implied by the model then robust sandwich variance estimators may be used [10, 12, 13]. The crucial step in order to do mediation analysis is to recover estimated covariances between parameter estimates from the different marginal model fits: this may be achieved by means of the marginal models approach [14–16]. Finally, the delta method may be used to derive estimated standard errors of any derived parameters of interest such as mediated effects and proportions mediated (see below) [17, 18]. An implementation in **R** [19] is provided in the **R** package *mmmVcov* available on github.

## Mediation analysis

The classical single-mediator approach is based on two generalized linear models of the following particular form [3]:

$$\begin{array}{lll} M_{1}:E\left(y_{i}\right) & = & g\left(\beta_{T_{1}}+x_{A,i}^{t}\beta_{A,1}\right)\\ M_{2}:E\left(y_{i}\right) & = & g\left(\beta_{T_{2}}+x_{M,i}^{t}\beta_{M_{1}}+x_{A,i}^{t}\beta_{A,2}\right). \end{array}$$ (1)

The parameters $\beta_{T_{j}}$ (*j* = 1, 2) (with the subscript *T*) are interpreted as total and direct effects of the treatment contrast of interest, respectively. The terms *x*_*M*,*i*_ and *x*_*A*,*i*_ (with subscripts *M* and *A*, respectively) are the parts of the design matrix corresponding to the mediator effect and additional covariates and the intercept, respectively. The corresponding parameters are denoted *β*_*M*,*j*_ and *β*_*A*,*j*_ with subscript referring to the models *M*_1_ and *M*_2_.

The above formulation in Eq (1) allows both categorical and continuous mediators. In case the mediator is a continuous, one-dimensional variable, *z*_*i*_ say, one additional model may be considered:

$$\begin{array}{c} M_{3}:E\left(z_{i}\right)=g\left(\beta_{T_{3}}+x_{A,i}^{t}\beta_{A,3}\right) \end{array}$$ (2)

where the parameter $\beta_{T_{3}}$ may be interpreted as the indirect effect contributing to the treatment contrast.

### Proportion mediated in logistic regression

Following Wang and Taylor (2002) [11] we considered two definitions for the scenario with binary mediator and outcome, allowing us to estimate the proportion mediated using the sandwich variance estimator. In this case Eqs (1) and (2) simplify to the following logistic regression models:

$$\begin{array}{ll} M_{1}: & \text{logit}\left\{Pr(T=1|Z=z)\right\}=\text{logit}\left\{\pi_{1}\left(z\right)\right\}=\beta_{T,1}z+\beta_{A,1}\\ M_{2}: & \text{logit}\left\{Pr(T=1|S=s,Z=z)\right\}=\text{logit}\left\{\pi_{2}\left(s,z\right)\right\}=\beta_{T,2}z+\beta_{M}s+\beta_{MT}sz+\beta_{A,2}\\ M_{3}: & \text{logit}\left\{Pr(S=1|Z=z)\right\}=\text{logit}\left\{\pi_{3}\left(z\right)\right\}=\beta_{T,3}z+\beta_{A,3} \end{array}$$

where *S*, *T*, and *Z* denote binary variables corresponding to the mediator, outcome, and treatment indicator. Model *M*_2_ is an interaction model. If the parameter *β*_*MT*_ is equal to 0 an additive model is obtained. The parameters *β*_*A*,1_, *β*_*A*,2_, and *β*_*A*,3_ are intercept terms; if additional covariates were included they would need to be averaged out in the definition given below. The first definition of the proportion mediated is simply the difference in log odds estimates divided by the log odds corresponding to the total effect:

$$\begin{array}{c} P=\left(\beta_{T_{1}}-\beta_{T_{2}}\right)/\beta_{T_{1}}. \end{array}$$

The second definition involves the difference on the probability scale. More specifically, the proportion mediated, *F*, is defined as follows:

$$\begin{array}{c} F=\frac{\left\{\pi_{3}\left(1\right)-\pi_{3}\left(0\right)\right\}\left\{\pi_{2}\left(1,0\right)-\pi_{2}\left(0,0\right)\right\}}{\pi_{1}\left(1\right)-\pi_{1}\left(0\right)} \end{array}$$

where 0/1 denotes absence or presence of mediator or treatment as appropriate. Note that the denominator is the total effect on the probability scale whereas the numerator is the part of the indirect effect (*π*_3_(1) − *π*_3_(0)) that may be attributed to the mediator. To obtain confidence intervals for these proportions mediated, when using marginal model fits, Wang and Taylor (2002) considered several approaches, one using Fieller’s theorem and two types of bootstrap approaches, all of which necessarily required an estimate of the correlation between parameter estimates from two models; this estimate was obtained assuming a joint model assuming multinomial distributions and then applying the delta method [11].

## A surrogate marker in ophthalmology

A randomized clinical trial in ophthalmology examined the effect of an experimental treatment, interferon-*α*, on loss of vision in patients with age-related macular degeneration [1, 2]. Patients aged 50 years or older with clinical signs of exudative age-related macular degeneration with subfoveal involvement were enrolled. Enrolment criteria included a best-corrected visual acuity in the eye under study of 20/320 or better with the use of the modified Early Treatment of Diabetic Retinopathy Study protocol and charts. The patients also needed an Eastern Coorporative Oncology Group performance status of 0 or 1. Exclusion criterias included chorooidal eovascularization greater than 12 Macular Photocoagulation Study disc areas in size and additional eye diseases that could compromise the visual acuity. Patients were randomized to either placebo or interferon-*α*-2a given in one of three doses three times a week for a year. Following Buyse and Molenberghs (1998) [1], we only consider the placebo group and the highest dose of interferon-*α*, 6 million international units. Visual acuity was assessed as the ability of reading lines of letters in decreasing size. Patients could receive credit for up till 17 lines and credits were given for the smallest line read with 0 or 1 error. The outcome considered was whether or not the patient had lost at least three lines of vision one year after baseline assessment. The mediator was whether or not at least two lines of vision were lost 6 months after baseline.

In this study data on three binary variables were recorded:

$$\begin{array}{lll} Z & = & \{\begin{array}{l} 0\;\text{if}\;\text{patient}\;\text{randomized}\;\text{to}\;\text{placebo},\\ 1\;\text{if}\;\text{patient}\;\text{randomized}\;\text{to}\;\text{interferon}-\alpha \end{array}\\ S & = & \{\begin{array}{l} 0\;\text{if}\;\text{patient}\;\text{had}\;\text{lost}\;\text{less}\;\text{than}\;\text{two}\;\text{letter}\;\text{lines}\;\text{of}\;\text{vision}\;\text{at}\;6\;\text{months}\\ 1\;\text{otherwise} \end{array}\\ T & = & \{\begin{array}{l} 0\;\text{if}\;\text{patient}\;\text{had}\;\text{lost}\;\text{less}\;\text{than}\;\text{three}\;\text{letter}\;\text{lines}\;\text{of}\;\text{vision}\;\text{at}\;1\;\text{year}\\ 1\;\text{otherwise} \end{array} \end{array}$$

In the placebo group 40 out of 104 patients had lost at least three lines of vision after one year. In the interferon-*α* group, 47 out of 86 patients lost at least three lines of vision. At 6 months, 17 out of the 104 and 68 out of 86 patients given placebo and interferon-*α*, respectively, had lost two lines of vision [1].

We fitted the logistic regression models *M*_1_, *M*_2_, and *M*_3_, which were previously described, using **R** [19]; the **R** lines are provided as supporting material (see S1 File).

The estimated total effect of the interferon-*α* treatment, expressed as an odds ratio, was 1.93 [1.08, 3.46] (based on *M*_1_). The direct effect of the treatment based on the additive model including the mediator corresponded to an odds ratio of 1.44 [0.68, 3.02] (based on *M*_2_ with *β*_*MT*_ = 0) and the effect of the treatment on the mediator corresponded to an odds ratio of 2.01 [1.13, 3.61] (based on *M*_3_).

The estimated mediated effects and proportions mediated based on differences on log odds scale and probability scale, respectively, along with their 95% confidence intervals are shown in Table 1; proportions mediated range from 0.45 to 0.69, such that roughly 50% of the treatment effect was mediated through visual acuity already at six months. The proportion mediated based on probabilities (*F*) was calculated both for the interaction and additive models, which were, however, not significantly different (*p* = 0.39).

**Table 1:** Estimated mediated effects and proportions mediated for the ophthalmology data example.

Measure of mediated effect	Estimate		Method for confidence interval		
	Effect	Prop.	Delta method	Fieller’s theorem	Bias-corrected bootstrap
*Log odds scale—P*					
Absolute	0.29		[-0.17, 0.75]		
Relative (proportion)		0.45	[-0.36, 1.25]	[-0.30, 4.35]	[-0.31, 4.25]
*Probability scale—F*					
*Interaction model*					
Relative (proportion)		0.69	[0.26, 1.12]	[0.17, 3.12]	[0.21, 3.11]
*Additive model*					
Absolute	0.11		[0.02, 0.20]	–	–
Relative (proportion)		0.65	[0.14, 1.17]	–	–

Estimated mediated effects and proportions mediated with corresponding 95% confidence intervals for two definitions and three methods for deriving confidence intervals (results for Fieller’s theorem and the bias-corrected bootstrap are taken from Wang and Taylor (2002) [11]).

## Discussion

We have reported the results of applying sandwich variance estimators in combination with the delta method in the context of mediation analysis in ophthalmology. The approach is robust against both model misspecification and unspecified correlation structures. We used marginal logistic regression model fits to recover correlations between parameter estimates rather than using bootstrap techniques or fitting a joint model, but still allowing for an asymptotically correct recovery of correlated information and not only bounding the information [20]. Specifically, we demonstrated the usefulness in the context of mediation analysis and surrogate markers: In the example three logistic regression models were fitted to binary outcomes and the proportion mediated through a surrogate marker was derived by combining estimates from these three models.

As pointed out by Wang and Taylor [11], the variability is less for the probability scale-based estimates than for the log odds-based estimates, and our results confirmed this finding, both for the interaction and additive model. The different methods for calculating confidence intervals, however, resulted in some differences: While the lower limits of the confidence intervals of the proportions mediated were similar across all three different methods, the upper limits disagreed substantially. The two methods used by Wang and Taylor (2002) [11] resulted in fairly similar upper limits as they were based on the same estimated correlation. Moreover, the resulting confidence intervals were asymmetric, most likely reflecting the right-skewed finite-sample distribution of the estimated proportion mediated. The Wald-type confidence interval based on the delta method, which is based on asymptotic results, is by definition symmetric and therefore it fails to pick up the asymmetry in the distribution of the estimated proportion mediated. However, agreement between approaches will improve as sample size increases. Likewise, upper limits of the confidence intervals above 1 will also disappear with increasing sample size as then the uncertainty on the derived parameter estimates will reduce. In practice, however, all the reported above upper limits would simply become equal to 1 by truncation.

The marginal models approach may also be useful for carrying out more complex types of mediation analysis [21], i.e., mediation analysis in observational studies where adjustment for covariates is important and where focus may be on multiple mediators. One such application using linear mixed models used for assessing mediated effects of socio-economic differences in cardio-metabolic risk markers has been reported previously by [22]. Specifically, these authors fitted linear mixed models of the form:

$$\begin{array}{ll} M_{1}: & y=X_{T}\beta_{T_{1}}+X_{A}\beta_{A_{1}}+Zb_{1}+\varepsilon_{1}\\ M_{2}: & y=X_{T}\beta_{T_{2}}+X_{M}\beta_{M_{2}}+X_{A}\beta_{A_{2}}+Zb_{2}+\varepsilon_{2} \end{array}$$

where the random effects in both models (*b*_1_ and *b*_2_) were bivariate normal distributed with mean 0 and a diagonal variance-covariance matrix (reflecting the hierarchical data structure with children in classes nested within schools). Subsequently, estimated mediated effects for individual and groups of potential mediators with 95% confidence intervals were derived [22].

In conclusion, we have demonstrated that an asymptotically based approach is available for inferences from several separately fitted logistic regression models used for mediation analysis. In the example analyzed we found qualitatively similar results for the asymptotic approach as for a previously proposed bootstrap approach. In general, small sample sizes could, however, lead to too low coverage when using the asymptotic approach; this aspect warrants further investigation. The asymptotic approach is an attractive alternative to specifying a joint model, which requires more, partly unverifiable assumptions on the correlation structure and is more prone to computational problems.

## Supporting information

S1 File R code to reproduce the example. (PDF)
