# Supplementary material for: Mediation analysis for logistic regression with interactions: Application of a surrogate marker in ophthalmology
Source: PLoS One. 2018 Feb 12;13(2):e0192857. doi: 10.1371/journal.pone.0192857 (PMC5809055; doi:10.1371/journal.pone.0192857)
Supplement: S1 File — (PDF) [file pone.0192857.s001.pdf]

Mediation Analysis for Logistic Regression with Interactions: Application of a Surrogate Marker in Ophthalmology

Supporting Information S1

Signe M. Jensen, Hanne Hauger and Christian Ritz

R code used in the example

```
# Loading required packages
library(car) # Version 2.1-4
library(devtools)
install_github("SigneMJensen/mmmVcov")
library(mmmVcov)


# Entering the data (as given in the paper)
sVec <- rep(c(0, 1, 0, 1), c(65, 38, 40, 47))
zVec <- rep(c(0, 1), c(103, 87))

y00 <- rep(c(0, 1), c(56, 9))
y01 <- rep(c(0, 1), c(8, 30))
y10 <- rep(c(0, 1), c(31, 9))
y11 <- rep(c(0, 1), c(9, 38))
yVec <- c(y00, y01, y10, y11)

# Fitting logistic regression models
model.tot <- glm(yVec ~ zVec, family = binomial)
model.dir <- glm(yVec ~ sVec + zVec, family = binomial)
model.med <- glm(sVec ~ zVec, family = binomial)

summary(model.tot)
summary(model.dir)
summary(model.med)

## Obtaining the doubly robust sandwich variance-covariance matrix
sandwich.vcov <- mjust(list(model.tot, model.tot, model.dir, model.dir, model.dir,
                            model.med, model.med),
                       list("(Intercept)", "zVec", "(Intercept)", "sVec", "zVec",
                            "(Intercept)", "zVec"))[["covar"]]

sandwich.vcov

## Combining parameter estimates
parmVec <- c(coef(model.tot), coef(model.dir), coef(model.med))
names(parmVec) <- c("beta10", "beta11", "beta20", "beta21", "beta22", "beta30", "beta31")
parmVec


## P
```

```
## Mediated effect
P.med.eff <- deltaMethod(parmVec, "beta11-beta22", sandwich.vcov)
P.med.eff
# P=0.29, CI: -0.17, 0.75

## Proportion mediated
Pval <- deltaMethod(parmVec, "1 - beta22/beta11", sandwich.vcov)
Pval
# P=0.44, CI: -0.36, 1.25


## Additive model

## F
## Mediated effect
F.med.eff <- deltaMethod(parmVec, "((exp(beta30+beta31)/(1+exp(beta30+beta31)) - exp(beta30)/(1+exp(beta30)))*
(exp(beta20+beta21)/(1+exp(beta20+beta21))-exp(beta20)/(1+exp(beta20))))", sandwich.vcov)
F.med.eff
# F=0.11, CI: 0.02, 0.20

## Proportion mediated
Fval <- deltaMethod(parmVec, "((exp(beta30+beta31)/(1+exp(beta30+beta31)) - exp(beta30)/(1+exp(beta30)))*
(exp(beta20+beta21)/(1+exp(beta20+beta21))-exp(beta20)/(1+exp(beta20))))/
(exp(beta10+beta11)/(1+exp(beta10+beta11)) - exp(beta10)/(1+exp(beta10)))", sandwich.vcov)
Fval
# F=0.65, CI: 0.14, 1.17


## Interaction model
szVec <- interaction(sVec, zVec)
model.dir2 <- glm(yVec ~ szVec-1, family = binomial)
summary(model.dir2)
anova(model.dir, model.dir2)  # p=0.39

## Combining parameter estimates
parmVec2 <- c(coef(model.tot), coef(model.dir2), coef(model.med))
names(parmVec2) <- c("beta10", "beta11", "beta200", "beta210", "beta201", "beta211", "beta30", "beta31")
parmVec2

sandwich.vcov2 <- mjust(list(model.tot, model.tot, model.dir2, model.dir2, model.dir2, model.dir2,
                            model.med, model.med),
                       list("(Intercept)", "zVec", "szVec0.0", "szVec0.1", "szVec1.0", "szVec1.1",
                            "(Intercept)", "zVec"))[["covar"]]

sandwich.vcov2


## Mediated effect
F.med.eff2 <- deltaMethod(parmVec2, "((exp(beta30+beta31)/(1+exp(beta30+beta31)) - exp(beta30)/(1+exp(beta30)))*
(exp(beta210)/(1+exp(beta210))-exp(beta200)/(1+exp(beta200))))", sandwich.vcov2)
F.med.eff2
# F=0.11, CI: 0.02, 0.21

## Proportion mediated
Fval2 <- deltaMethod(parmVec2, "((exp(beta30+beta31)/(1+exp(beta30+beta31)) - exp(beta30)/(1+exp(beta30)))*
(exp(beta210)/(1+exp(beta210))-exp(beta200)/(1+exp(beta200))))/
(exp(beta10+beta11)/(1+exp(beta10+beta11)) - exp(beta10)/(1+exp(beta10)))", sandwich.vcov2)
Fval2
# F=0.69, CI: 0.26, 1.12
```
